# Case study: painful, red eye in a Ugandan farmer

**Published:** 2022-09-20

**Authors:** Simon Arunga, Abel Ebong, Francis Orishaba

**Affiliations:** Clinical Lecturer and Ophthalmologist: Mbarara University and Regional Referral Hospital Eye Centre, Mbarara, Uganda.; Ophthalmologist: Mbarara University of Science and Technology.; Microbiologist: Mbarara University of Science and Technology.

## History

A 74-year-old male Ugandan farmer presented to a referral hospital with a 25-day history of a painful, red left eye with blurred vision and tearing. He reported that, four days before the pain started, he had been on the farm spraying his cattle. There was no clear history of trauma and he did not use contact lenses. There was no other relevant past ophthalmic history.

**Treatment history.** After developing the above symptoms, the patient reported that he had started using unknown eye drops which he purchased from the local pharmacy, as well as traditional plant-based eye medicines.

**Medical history.** He reported that he was HIV negative and had no history of diabetes or any chronic illness. He was not taking any systemic medication.

**Figure 1:**
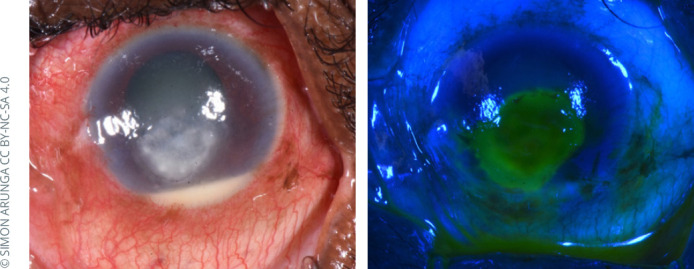
Corneal photos, on presentation.

**Question 1**

**What clinical signs do you see?** *(Tick all that apply)*

- Conjunctival hyperaemia
- Hypopyon
- Corneal infiltrate
- Satellite lesions
- Epithelial defect

## Examination

On the day of admission (day 0), the left visual acuity was perception of light (PL), with no improvement on pinhole. There was a white corneal infiltrate measuring 6.0 mm by 4.5 mm (Figure 1), an overlying epithelial defect of 5.5 mm × 5.0 mm, 70% corneal thinning, and a 1.5 mm hypopyon. Other than an unaided visual acuity of 6/36 due to cataract, examination of the right eye was normal.

**Question 2**

**What is your working diagnosis?** *(Choose 1 option)*

- Fungal keratitis
- Bacterial keratitis
- Viral keratitis
- Traumatic ulcer

**Question 3**

**What investigations could you perform?** *(Tick all that apply)*

- Corneal scrape for microscopy and culture
- PCR swab test
- In vivo confocal microscopy
- Antimicrobial susceptibility testing of cultured isolates

## Investigations

**Figure 2:**
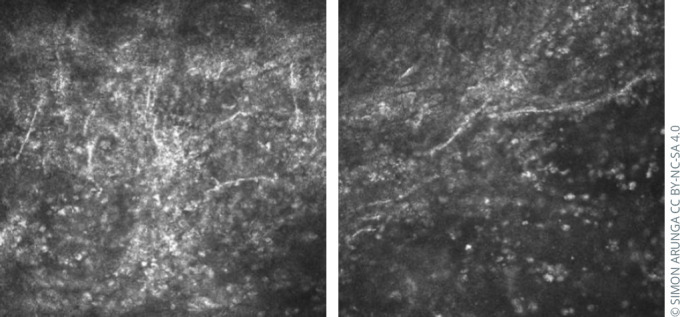
Baseline confocal microscope pictures.

In vivo confocal microscopy was performed, and fungal hyphae were seen (Figure 2). Corneal tissue samples were collected for microscopy (Gram stain, potassium hydroxide (KOH), and calcofluor white (CFW) preparations), and inoculated into the following culture media: blood agar, chocolate agar, potato dextrose agar (PDA), and brain heart infusion broth. The initial Gram stain, CFW, and KOH slides revealed fungal hyphae. A blood sample was also drawn to test for HIV and diabetes, which are known risk factors for microbial keratitis.

**Figure 3:**
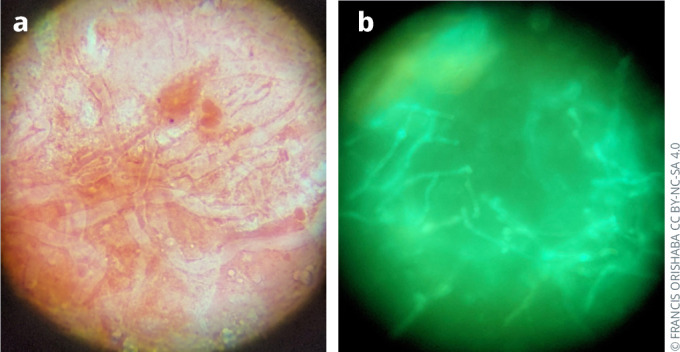
**(a)** Gram stain and **(b)** calcofluor white stained preparations of corneal tissue.

**Figure 4:**
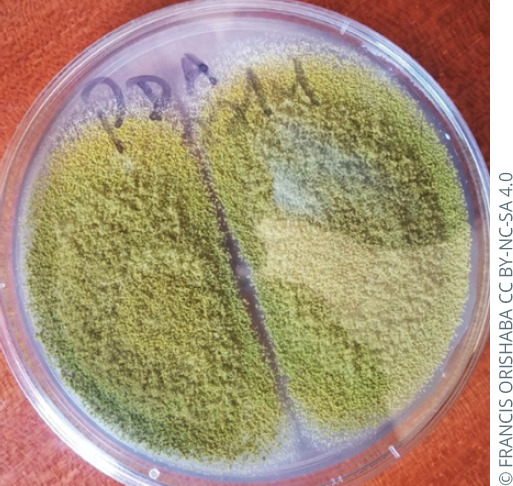
Fungal culture on potato dextrose agar at 7 days.

Fungal growth (*Aspergillus* sp.) was observed on PDA within 1 week (Figure 4). Blood results were negative for HIV and blood glucose levels were normal.

**Question 4**

**How would you treat this patient?** *(Tick all that apply)*

- Antiviral eye ointment, e.g., aciclovir eye ointment 5 times a day
- Antifungal eye drops such as topical natamycin 5%, chlorhexidine 0.2%, voriconazole 1%, or amphotericin B 0.05% hourly, depending on local availability
- Steroid eye drops, e.g., prednisolone 1%
- Cycloplegic eye drops, e.g., atropine 1%
- Antibiotic eye drops, e.g., ciprofloxacin 0.3%

## Management

The patient was started on hourly antifungal eye drops (either natamycin 5% with chlorhexidine 0.2%, or just natamycin 5% – the exact agent is still masked to the clinical team as the patient is part of an ongoing randomised controlled clinical trial) as well as topical ciprofloxacin 0.3% four times a day, and atropine 1% eye drops. Exactly 48 hours after initiation of treatment, the patient was reporting improvement as manifested by a decrease in pain and tearing. The infiltrate measured 4.2 mm by 3.0 mm (Figure 4) and the epithelial defect measured 4.6 mm by 4.0 mm. The hypopyon was slightly smaller.

**Figure 5:**
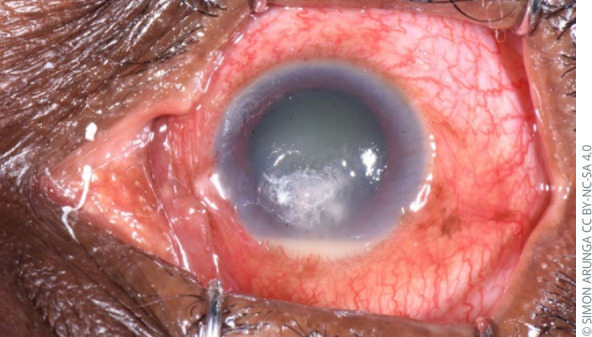
Corneal photo after 48 hours of treatment.

## Dramatic worsening

**Figure 6:**
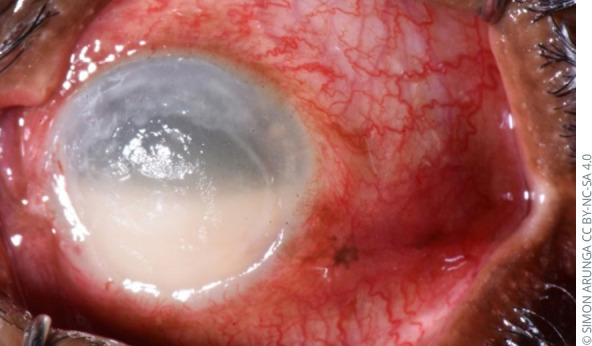
Corneal photo at day 7, showing a very large hypopyon.

After 7 days, the patient returned for a scheduled review. The visual acuity in the affected eye was perception of light (PL). The corneal infiltrate had increased in size and now measured 11.0 mm × 10.0 mm (Figure 5), with an overlying epithelial defect measuring 8.5 mm × 8.2 mm (Figure 6) with 70% corneal thinning and a 5 mm hypopyon.

**Figure 7:**
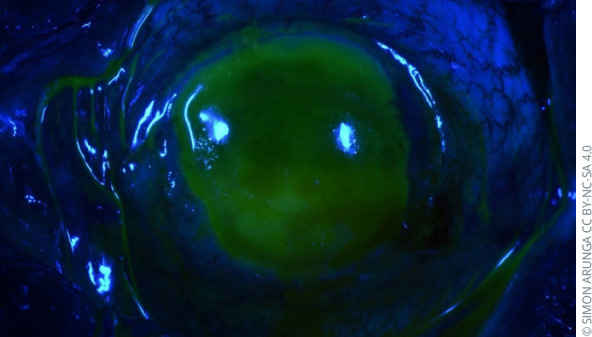
Stained corneal picture at day 7, showing an epithelial defect over almost the entire cornea.

**Figure 8:**
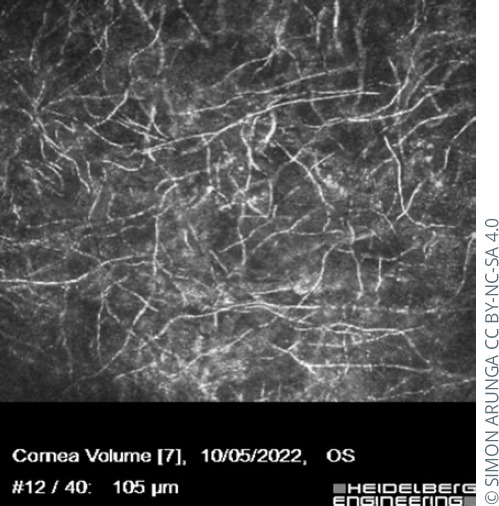
IVCM picture at day 7, showing increased fungal density.

**Question 5**

**What are the possible reasons for the patient's worsening condition?** *(Tick all that apply)*

- The patient did not adhere to treatment
- A secondary bacterial infection has developed
- Development of microbial resistance

## Further management

One of the key things to check here would be adherence to treatment. Fungal keratitis requires intensive and prolonged treatment and any lapse or pause in the application of eye medication will allow an infection to continue/progress. Concomittent bacterial infection can also be associated with disease progression. In this particular case microbiology results were negative for bacteria at 7-day follow-up. Microbial resistance to treatment might need to be considered, but it would not be expected to develop in such a short time period, unless the causative organism is intrinsically resistant to the antimicrobial treatment being administered. If antimicrobial susceptibility testing is available this will help to guide treatment choice. At this stage, it would be important to take further samples for microbiological investigation and reassess treatment options. The patient should be admitted so that intensive treatment can be monitored. As well as the initial antifungal treatment, additional treatment such as amphotericin B 0.05% could be used, as well as antibiotic drops. The patient needs to be made aware that there is a significant risk of losing the eye and that an artificial eye may be needed.

## Reflection

Microbial keratitis is a common presentation, but it brings many challenges on different levels. Patients may use traditional eye medicines which are often of non-sterile origin, or steroid eye drops from a pharmacy, which can cause the infection to worsen. Presentation is often delayed, and by the time patients are seen, they have often already developed a very advanced infection which is not responsive to treatment. It is important to determine the type of organism causing the infection, however, diagnostic microbiology services may not be available. Natamycin is considered first-line treatment for fungal keratitis, but it is not available in many countries. Evidence is emerging for the use of chlorhexidine where natamycin is not available.^4^

## ANSWERS

1. **All are correct.**
2. **a is most likely.** The presence of an epithelial defect with underlying infiltrates and a hypopyon with hyperaemia are typical of microbial keratitis (corneal infection). The presence of satellite lesions and the delayed presentation are suggestive of fungal keratitis.^1^ In tropical latitudes fungal keratitis is also very common.^2^ Based on clinical signs alone however, it is not possible to rule out the possibility of a bacterial infection, or a mixed fungal-bacterial infection.
3. **All are correct.** All are correct. It is very important to determine what organism(s) are causing the infection so that treatment can be targeted appropriately.^3^
4. **b.** Antifungal eye drops must be started, with natamycin being first line if available. These should be given intensively, e.g., every hour day and night for the first two days and then reviewing. Antibiotic eye drops would also be reasonable as there may be bacterial (co-)infection. Cycloplegic eye drops will help the patient feel more comfortable and should be used. Steroid eye drops are contraindicated for active fungal keratitis as they can cause the infection to worsen. The presentation is not suggestive of a viral infection.
5. **a and b.** Microbial resistance to treatment might need to be considered, but it would not be expected in such a short time period.
